# A systems biology analysis of brain microvascular endothelial cell lipotoxicity

**DOI:** 10.1186/1752-0509-8-80

**Published:** 2014-07-04

**Authors:** Hnin H Aung, Athanasios Tsoukalas, John C Rutledge, Ilias Tagkopoulos

**Affiliations:** 1 Division of Cardiovascular Medicine, Department of Internal Medicine, University of California, Davis, CA 95616, USA; 2 UC Davis Genome Center, University of California, Davis, CA 95616, USA; 3 Department of Computer Science, University of California, Davis, CA 95616, USA

**Keywords:** Activating transcription factor 3, Microarray, Triglyceride-rich lipoprotein, Blood–brain barrier

## Abstract

**Background:**

Neurovascular inflammation is associated with a number of neurological diseases including vascular dementia and Alzheimer’s disease, which are increasingly important causes of morbidity and mortality around the world. Lipotoxicity is a metabolic disorder that results from accumulation of lipids, particularly fatty acids, in non-adipose tissue leading to cellular dysfunction, lipid droplet formation, and cell death.

**Results:**

Our studies indicate for the first time that the neurovascular circulation also can manifest lipotoxicity, which could have major effects on cognitive function. The penetration of integrative systems biology approaches is limited in this area of research, which reduces our capacity to gain an objective insight into the signal transduction and regulation dynamics at a systems level. To address this question, we treated human microvascular endothelial cells with triglyceride-rich lipoprotein (TGRL) lipolysis products and then we used genome-wide transcriptional profiling to obtain transcript abundances over four conditions. We then identified regulatory genes and their targets that have been differentially expressed through analysis of the datasets with various statistical methods. We created a functional gene network by exploiting co-expression observations through a guilt-by-association assumption. Concomitantly, we used various network inference algorithms to identify putative regulatory interactions and we integrated all predictions to construct a consensus gene regulatory network that is TGRL lipolysis product specific.

**Conclusion:**

System biology analysis has led to the validation of putative lipid-related targets and the discovery of several genes that may be implicated in lipotoxic-related brain microvascular endothelial cell responses. Here, we report that activating transcription factors 3 (ATF3) is a principal regulator of TGRL lipolysis products-induced gene expression in human brain microvascular endothelial cell.

## Background

The estimated prevalence of dementia of persons greater than 70 years of age is 14.7% [1]. The yearly cost attributable to dementia is between $43,000/patient and $70,000/patient and the total monetary cost of dementia in 2010 was between $157 billion and $215 billion. Add to this, the enormous personal and emotional cost to, not only the patient, but also to family, friends, and co-workers, and we have a national tragedy that is about to unfold as the baby boomers transition to the elderly. These financial and personal costs place dementia on par with the costs attributable to ASCVD and cancers.

**Figure 1:**
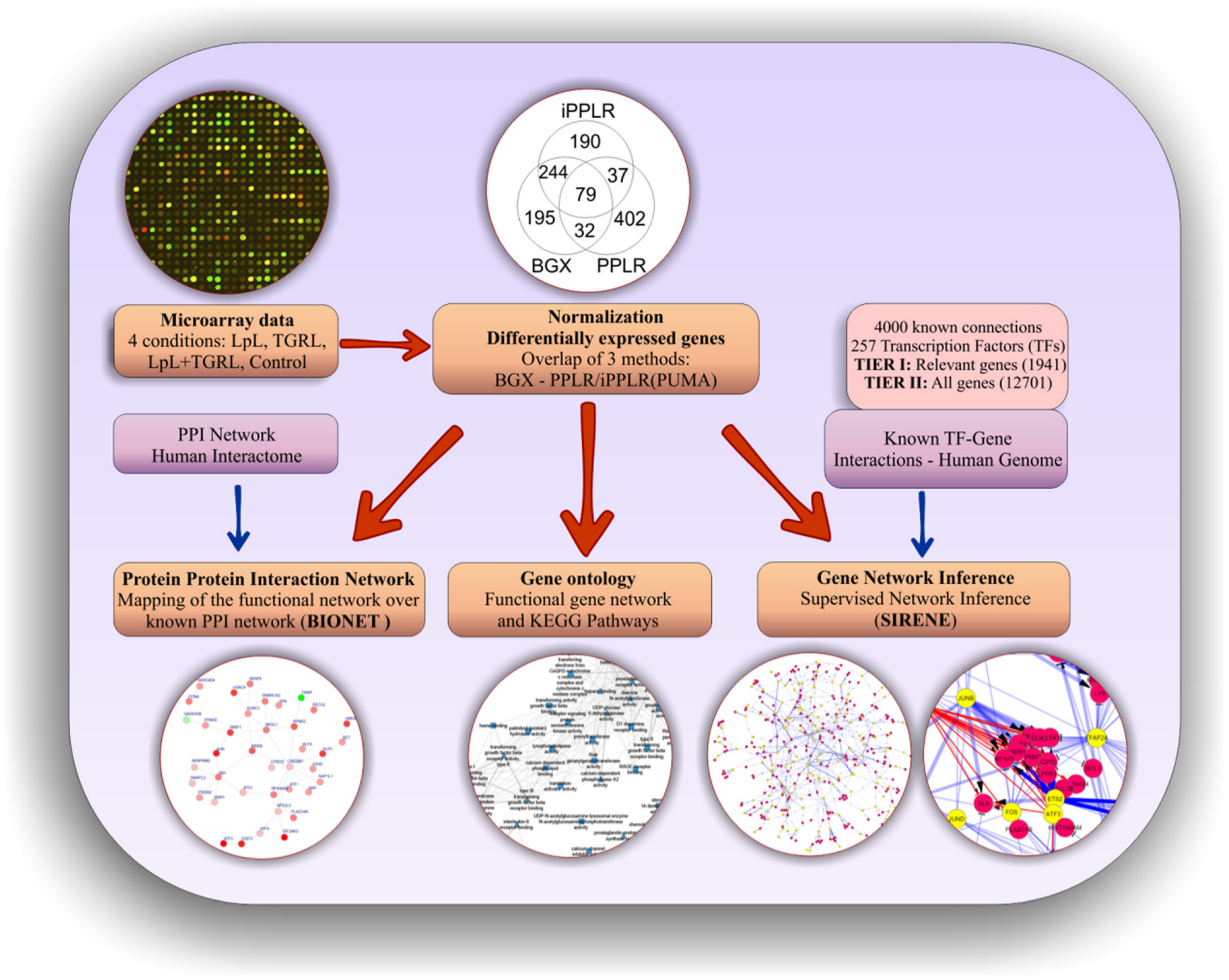
**Overview of the experimental and computational procedures.** Expression profiles of endothelial cells were taken after treatment in four environments and their expression was normalized by using three probabilistic methods that exploit the difference in the distributions of the perfectly matched and mismatched probes. Then we built the gene ontology and functional networks that we overlapped with the known PPI network, which is available in literature, to identify implicated processes and genes. To uncover putative TF-gene interactions that take place under these conditions, we used a supervised network inference approach that utilizes known information about TF genes and their targets to find similar expression patterns in our data and thus uncover novel interactions. Finally, top ranked over/under expressions are validated by qRT-PCR and their relative levels are quantified.

One of the potential inducers of neurovascular inflammation is triglyceride-rich lipoprotein (TGRL) particles and their lipolysis products [2]. Lipoprotein lipase (LpL) is anchored to the brain microvascular endothelium, where it binds and hydrolyzes TGRL particles to smaller lipolysis products, such as fatty acids [3]. TGRL lipolysis products are generated at the luminal surface of the vascular endothelium and the lipolysis products in high physiological and pathophysiological concentrations can potentially injure the endothelium directly, increase the permeability of the blood–brain barrier (BBB), and/or injure astrocytes and neurons within the brain. Our studies have shown that TGRL lipolysis products have a dramatic effect on endothelial cell injury, which is of much greater magnitude than TGRL particles, such as chylomicrons and VLDL [4].To explore neurovascular lipotoxicity further, we treated human brain microvascular endothelial cell (HBMVEC) with TGRL lipolysis products and then we used genome-wide transcriptional profiling to obtain transcript abundances over four conditions. We then identified regulatory genes and their targets that have been differentially expressed (DE) through analysis of the datasets with various statistical methods (Figure 1). We created a functional gene network by exploiting co-expression observations through a guilt-by-association assumption. Concomitantly, we used various network inference algorithms to identify putative regulatory interactions and we integrated all predictions to construct a consensus gene regulatory network that is TGRL lipolysis product specific. This analysis has led to the validation of putative lipid-related targets and the discovery of several genes that may be implicated in TGRL lipolysis-related lipid response. Our system biology analysis also identified that activating transcription factors 3 (ATF3) is a principal regulator and induces expression of downstream inflammatory response genes in HBMVEC treated with TGRL lipolysis products.

## Results

**Figure 2:**
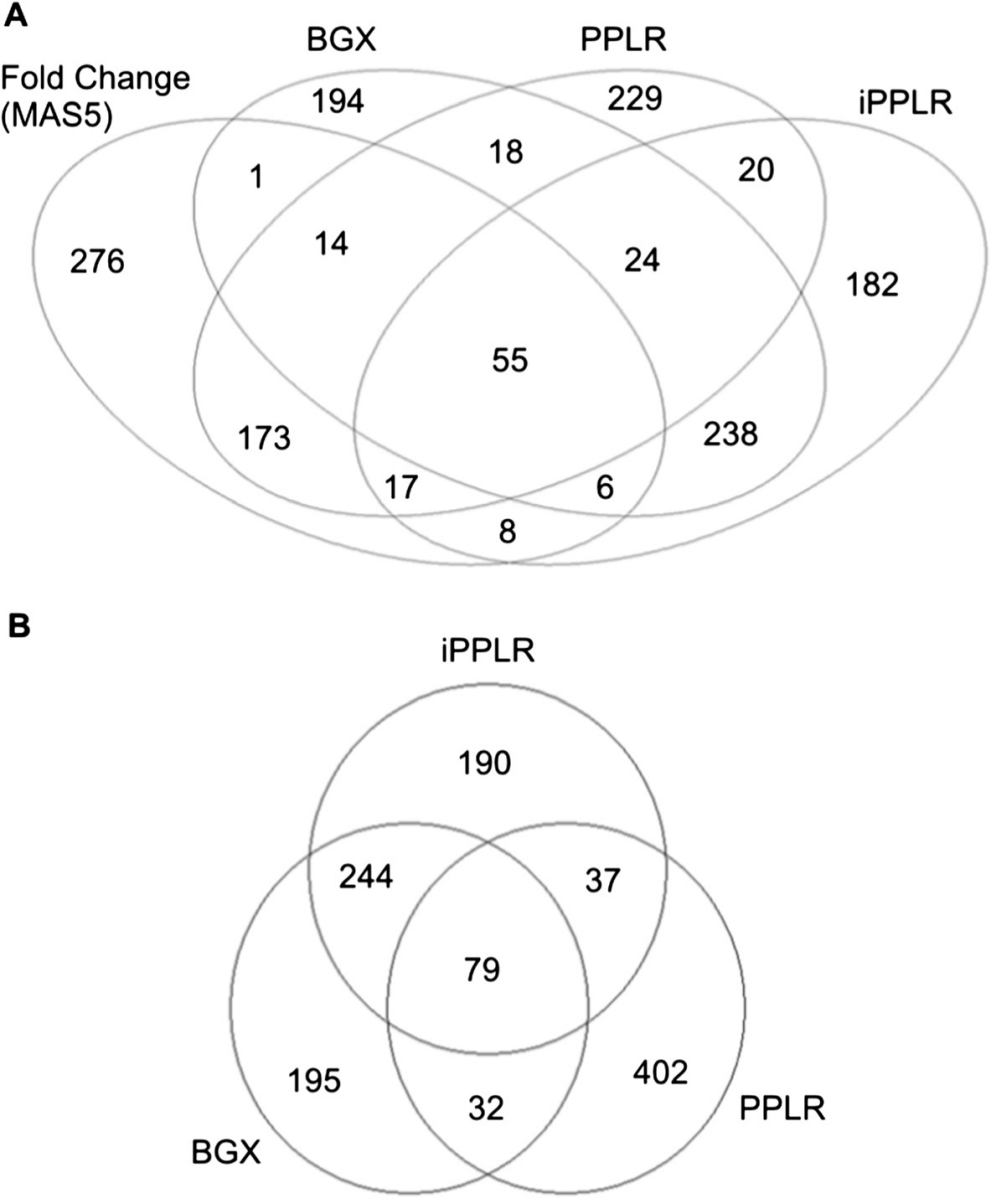
**Venn diagrams on differentially expressed (DE) genes. (A)** The most highly-ranked 550 genes from all available methods; **(B)** Venn diagram shows overlap for probabilistic-only methods that were used to identify the consensus list of DE genes. The gene cut-off (550 genes) corresponds approximately to a p-value of 0.05 or lower in all methods.

As shown in Figure 2, there is some overlap between the different DE techniques used, as well as differences in the top gene candidates identified, due to the different features and underlying statistical assumptions of each technique. Table 1 depicts the top 20 genes that have the highest DE between the treatment with the combination of TGRL and LpL (TGRL lipolysis products), and the control (media-only) that are within the statistical cut-off value (p-value < 10^-4^), over all techniques. Consistent with our previous results in endothelial cells [5], ATF3 shows clear up-regulation after TGRL lipolysis product treatment.

**Table 1:** Top 20 differentially expressed (DE) genes (consensus over three methods); Numbers denote the fold-change (FC) between the TGRL + LpL condition and the control (media-only)

**GENE SYMBOL**	**GENE NAME**	**iPPLR FC**	**BGX FC**	**PPLR FC**	**p-values**
ATF3	Activating transcription factor 3	1,86	2,67	3,06	10^-14^
BHLHE40	Basic helix-loop-helix family, member e40	0,35	0,23	0,32	10^-9^
PRNP	Prion protein	1,47	3,09	3,89	10^-8^
CSGALNACT2	Chondroitin sulfate N-acetylgalactosaminyltransferase 2	1,48	3,90	4,36	10^-8^
ADD3	Adducin 3 (gamma)	1,61	4,29	4,52	10^-7^
MAD2L1	MAD2 mitotic arrest deficient-like 1 (yeast)	1,07	3,49	3,31	10^-7^
ADAM9	ADAM metallopeptidase domain 9	1,22	3,19	3,38	10^-7^
ZNF217	Zinc finger protein 217	1,83	6,04	4,94	10^-7^
DDIT3	DNA-damage-inducible transcript 3	1,11	2,50	2,83	10^-6^
STK39	Serine threonine kinase 39	1,13	2,94	3,81	10^-6^
LIMS1	LIM and senescent cell antigen-like domains 1	1,14	3,03	3,10	10^-6^
MMRN1	Multimerin 1	1,62	2,95	3,34	10^-5^
CSGALNACT2	Chondroitin sulfate N-acetylgalactosaminyltransferase 2	1,06	3,99	3,63	10^-5^
PAIP1	Poly(A) binding protein interacting protein 1	1,04	2,34	2,80	10^-5^
HDGFRP3	Hepatoma-derived growth factor, related protein 3	1,05	2,26	2,84	10^-5^
HES1	Hairy and enhancer of split 1, (Drosophila)	0,56	0,34	0,42	10^-5^
FRMD4B	FERM domain containing 4B	1,13	2,80	2,92	10^-5^
DUSP6	Dual specificity phosphatase 6	1,08	3,03	2,71	10^-4^
PRKAR1A	Protein kinase, cAMP-dependent, regulatory, type I, alpha	1,12	2,69	2,73	10^-4^
TTC37	Tetratricopeptide repeat domain 37	1,13	2,72	2,48	10^-4^

**Figure 3:**
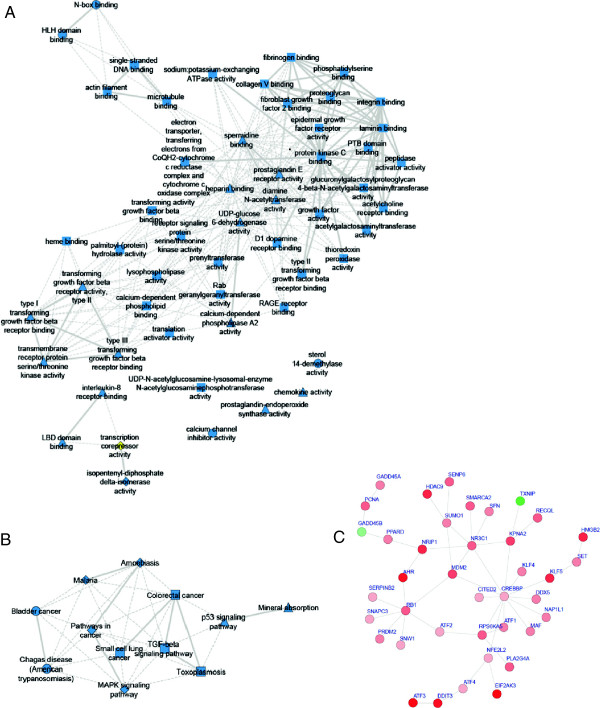
**Functional and PPI networks related to TGRL lipolysis treatment of endothelial cells. (A)** Functional gene network, **(B)** KEGG pathways and **(C)** PPI network. Color denotes differential expression ranging from strong down-regulation (green) to strong up-regulation (red). Solid/dashed lines are based on statistical significance (0.05).

**Figure 4:**
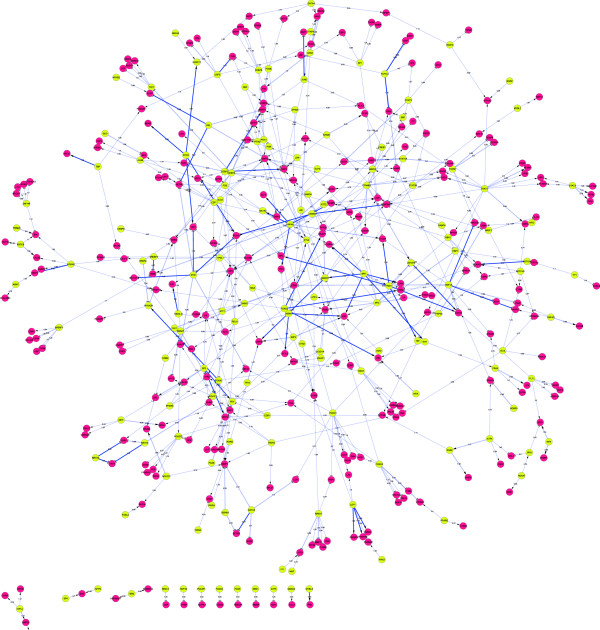
**TGRL lipolysis treatment network of differentially expressed genes.** Edges denote regulatory interactions (TF-DNA binding), yellow are transcription factor (TF) genes and red nodes are differentially regulated targets. Edge weight corresponds to the Pearson correlation coefficient ρ of the expression profiles in connected nodes and the value of the edge (see Additional file 1: Table S1) denotes how statistically significant the interaction is (in all cases p-value < 10^-2^). The resulting network has 151 TFs, 272 target genes (X and Y up- and down-regulated, respectively), 236 inhibitory interaction (ρ < -0.25), 265 activatory interactions (ρ > 0.25).

The 79 DE genes that have been in parallel identified from all methods form a functional gene network with 54 functional categories that are significantly over-represented in this gene set, which also implicates 12 KEGG pathways (Figure 3A). We integrated the expression-derived relationships with the known protein-protein interaction (PPI) network that was extracted by using the BIONET Bioconductor package [6] and the Interactome Library [7] (Figure 3C). In addition, we used a support vector machines (SVM) method called SIRENE [8] together with a dataset where we amassed of all known TF-gene interactions (~4000) in order to construct a condition-specific Gene Regulatory Network (GRN) associated with TGRL lipolysis treatment (Figure 4). The resulting network has 151 TFs, 272 target genes (X and Y up- and down-regulated, respectively), 236 inhibitory interactions (ρ < -0.25), and 265 activatory interactions (ρ > 0.25).

### Genome-wide analysis of TGRL lipolysis-treated HBMVEC

Differential analysis of gene expression data showed that HBMVEC treated with either LpL or TGRL altered the expression of a small percentage of genes (3.01% and 4.03%, respectively, when compared to control). TGRL + LpL (TGRL lipolysis products) treatment affected the expression of 5.61% of all genes detected (~14,500). Functional classification of genes modulated by lipolysis product (TGRL + LpL) treatment identified multiple functional classes including transcription factors, inflammatory responses, apoptosis, cell cycle, cell proliferation, ion metabolism, lipid metabolism, kinase activity, signalling pathways, DNA binding, protein binding, protein folding, and proteins of unknown function.

### Differentially expressed transcription factors

**Figure 5:**
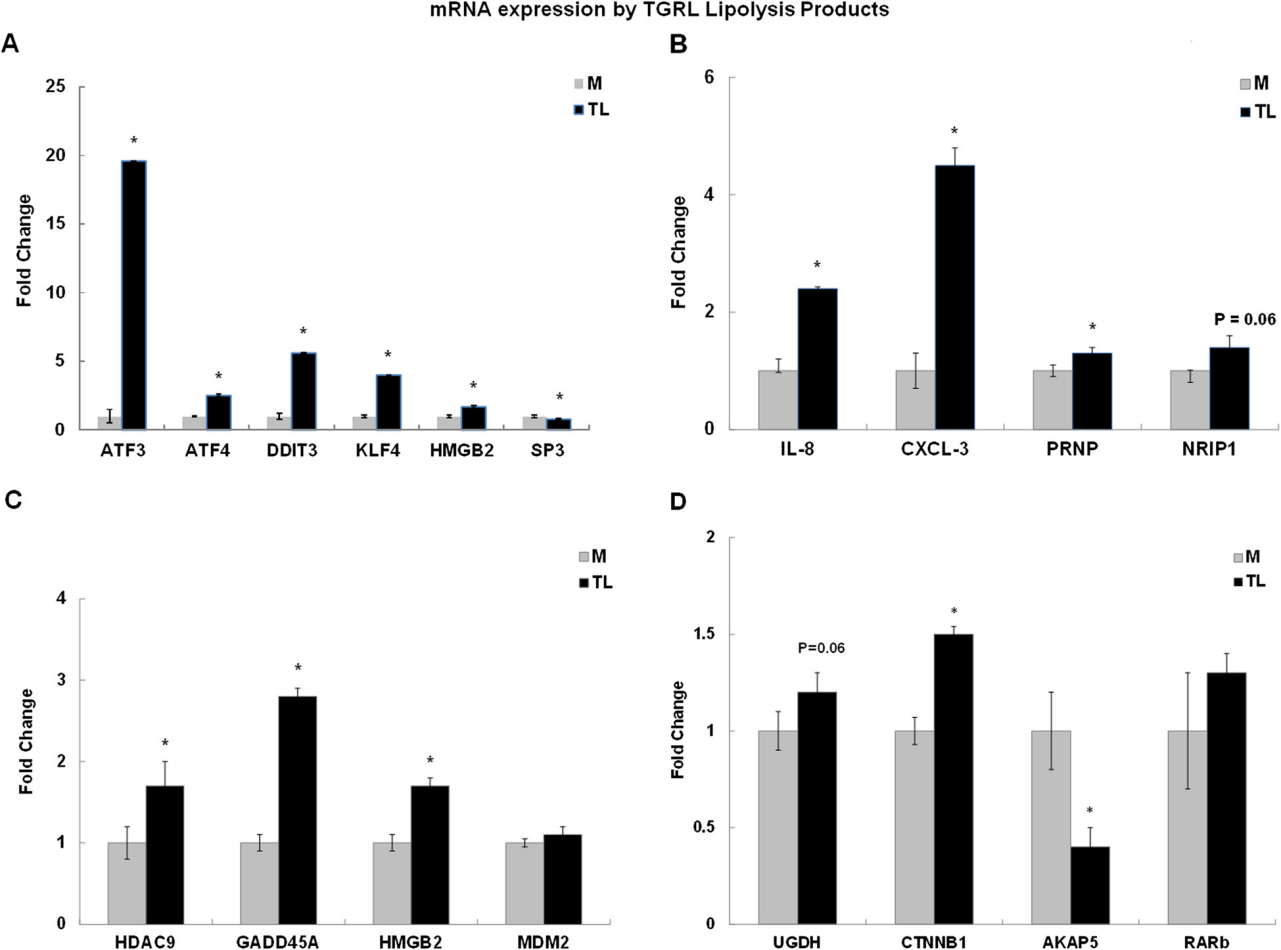
**Confirmation of selected genes by qRT-PCR.** TGRL lipolysis products (TL) compare to Media (M) alone treatment. Genes related to **(A)** Transcription factors, **(B)** Pro-inflammatory Response, **(C)** Cell cycle and Apoptosis, **D)** Metabolism and Signalling pathway. HBMVEC were treated with either Media (M) or TGRL lipolysis products (TL) for 3 hr. Microarray analysis: (pooled n = 3 per GeneChip as compared to TGRL-treated group). For qRT-PCR, the expression of each gene was normalized to that of GAPDH and then fold change was calculated as a ratio of expression after lipolysis products treatment relative to TGRL controls (individual n = 3). An asterisk (*) denotes p-value ≤ 0.05.

TGRL lipolysis products activated genes encoding transcription factors including activating transcription factor (ATF3), ATF4, ATF2, DNA damage-inducible transcript 3 (DDIT3), CREB binding protein (CREBBP), Krueppel-like factor 4 (KLF4), KLF5, aryl hydrocarbon receptor (AHR), peroxisome proliferator-activated receptor delta (PPARD). ATF3 is a member of the mammalian activation transcription factor/cAMP responsive element-binding (CREB) protein and a member of the ATF family of transcription factors. This gene is induced by a variety of signals including many of those encountered by cancer cells, and is involved in the complex process of cellular stress response [9] and post-translational modifications have been established. For instance, it has been reported previously that upon UV irradiation, two transcription factors, c-Jun and ATF2, are phosphorylated by the JNK/SAPK family of stress-induced kinases [10]. Many stress responses have been studied in tissue culture cells by using signals including UV irradiation [11], cytokines [12], and modulated by gadd153/Chop10 [13]. ATF4 encodes a transcription factor that was originally identified as a widely expressed mammalian DNA binding protein. Recently it has been found that ATF4 mediates hyperglycemia-induced endothelial inflammation and retinal vascular leakage in mouse [14]. Up-regulation of ATF3 (19.6-fold) and ATF4 (3.6-fold) by TGRL lipolysis in HBMVEC was confirmed by qRT-PCR (Figure 5A).

CREB binding protein (CREBBP) is ubiquitously expressed and is involved in the transcriptional coactivation of many different transcription factors and known to play critical roles in embryonic development, growth control, and homeostasis by coupling chromatin remodeling to transcription factor recognition. Transforming growth factor-β signalling pathways mediate epithelial-mesenchymal transition are dependent on the transcriptional co-activator CREBBP [15]. CREBBP was increased (1.1-fold) but not significantly up-regulated by TGRL lipolysis products (data not shown). DNA damage-inducible transcript 3 (DDIT3; 5.6-fold up-regulation; Figure 5A) is a member of the CCAAT/enhancer binding proteins (C/EBPs) (CHOP) family of transcriptional factors that regulate cell cycle and apoptosis.

Krueppel-like factor 4 (KLF4; 4-fold up-regulation; Figure 5A) is known to be endothelial Kruppel-like zinc finger protein. KLF4 differentially regulates pertinent endothelial targets and important regulators of vascular homeostasis and atherothrombosis [16]. Kruppel-like factor 4 regulates endothelial activation in response to pro-inflammatory stimuli [17]. Peroxisome proliferator-activated receptor delta (PPARD or PPARδ) (0.6-fold down-regulation; Figure 5A) is a member of the peroxisome proliferator-activated receptor (PPAR) family. PPARs mediate a variety of biological processes, and may be involved in the development of several chronic diseases, including diabetes, obesity, atherosclerosis, and cancer. PPARs are nuclear receptors regulating the expression of genes involved in lipid and glucose metabolism [18,19]. PPARD is an important regulator of fatty acid (FA) metabolism [20]. Increase expression of PPARD has been reported in hepatic steatosis that is induced by oleic acid. Several lines of evidence point to a negative regulatory role for PPARβ/δ in inflammatory responses of the skin. Thus, mice deficient for PPARβ/δ showed an increased inflammatory response to the topical application of O-tetradecanoylphorbol-13-acetate [21].

The Specific protein 3 gene (Sp3; 0.8-fold down-regulation; Figure 5A) encodes for transcription factors that regulate transcription by binding to consensus GC- and GT-box regulatory elements in target genes. Sp3 is an inducer of apoptosis and a marker of tumor aggressiveness [22].

### TGRL lipolysis products activate pro-inflammatory factors

Interleukin 8 (IL-8 or CXCL8; 2.4-fold up-regulation; Figure 5B), a member of the CXC chemokine family, is one of the major mediators of inflammatory responses. Chemokine ligand 3 (CXCL3; 4.5-fold up-regulation; Figure 5B), which is known to be induced by oxidized low-density lipoprotein, was also was found to be induced by TGRL lipolysis products. Prion protein (PRNP; 1.3-fold up-regulation; Figure 5B) is a plasma membrane glycosylphosphatidylinositol-anchored glycoprotein that tends to aggregate into rod-like structures. Recently, PRNP has been shown to mediate the toxicity of other pathological protein aggregates, including oligomers of the amyloid β (Aβ) peptide, which are associated with Alzheimer’s disease PRNP [23,24]. It has been reported that PRNP gene contributes the association between the methionine/valine (M/V) polymorphism and risk of Alzheimer disease [25].

Nuclear receptor interacting protein 1 (NRIP1 also known as RIP140; 1.4-fold up-regulation; Figure 5B), is a nuclear protein that specifically interacts with the hormone-dependent activation domain AF2 of nuclear receptors. NRIP1 is a key regulator that modulates transcriptional activity of a variety of transcription factors, including the estrogen receptor and has an important role in regulating lipid and glucose metabolism. Mice devoid of the co-repressor protein RIP140 are lean, show resistance to high-fat diet-induced obesity and hepatic steatosis, and have increased oxygen consumption [26]. Increased RIP140/NRIP1 level is associate with inflammation and disorders of lipid and glucose metabolism in diabetic patients [27].

SPEG (0.7-fold down-regulation; Figure 5B), also known as aortic preferentially expressed gene-1 (APEG-1), was down-regulated by TGRL lipolysis products. APEG-1 appears to be expressed only in highly differentiated aortic smooth muscle cells (ASMC) in normal vessel walls but it’s mRNA was down-regulated in dedifferentiated ASMC in response to vascular injury [28].

### TGRL lipolysis product-induced endothelial cell apoptosis

Histone deacetylase 9 (HDAC9; 1.7-fold up-regulation; Figure 5C) plays a critical role in transcriptional regulation, cell cycle progression, and developmental events. Histone acetylation/deacetylation alters chromosome structure and affects transcription factor access to DNA. Growth arrest and DNA-damage-inducible, alpha (GADD45A; 2.8-fold up-regulation; Figure 5C) and beta (GADD45B) genes are the first well-defined p53 downstream genes. They can be induced by multiple DNA-damaging agents and stressful growth arrest conditions, such as IR and UV radiation, and play important roles in the control of cell cycle checkpoint, DNA repair processes, and signalling transduction. The protein encoded by this gene responds to environmental stresses by mediating activation of the p38/JNK pathway via MTK1/MEKK4 kinase. No change in GADD45A and loss function of GADD45B suggested that its normal role in the pituitary includes acting as a brake to cell proliferation and survival [29].Despite their central position in the TGRL-centered PPI network, Mouse double minute 2 homolog (MDM2) and S100 calcium binding protein B (S100B) were not found to be differentially regulated in our samples (p-value > 0.05; Figure 5C). MDM2 is a target gene of the transcription factor tumor protein p53 and also affects the cell cycle, apoptosis, and tumorgenesis through interactions with other proteins. Over expression of this gene results in excessive inactivation of tumor protein p53, diminishing its tumor suppressor function. S100B is a member of the S100 family of proteins containing 2 EF-hand calcium-binding motifs. S100 proteins are localized in the cytoplasm and/or nucleus of a wide range of cells, and involved in the regulation of a number of cellular processes such as cell cycle progression and differentiation. The altered expressions of this gene have been implicated in several neurological, neoplastic, and other types of diseases, including Alzheimer’s disease.

In contrast, the High mobility group box 2 (HMGB2) was up-regulated 1.7-fold by TGRL lipolysis (Figure 5C). HMGB2 gene encodes a member of the non-histone chromosomal high mobility group protein families. In vitro studies have demonstrated that this protein is able to efficiently bend DNA and form DNA circles. HMGB2 is known to stabilize p53 in HeLa cells [30]. Overexpression of HMGB2 in hepatocellular carcinoma is associated with poor prognosis and tumor development [31].

### TGRL lipolysis product-induced metabolism and signalling pathways

UDP-glucose 6-dehydrogenase (UGDH, 1.2-fold up-regulation (p = 0.06); Figure 5D), the protein encoded by this gene converts UDP-glucose to UDP-glucuronate and thereby participates in the biosynthesis of glycosaminoglycans such as hyaluronan, chondroitin sulfate, and heparan sulfate. The expression of UGDH is up-regulated by transforming growth factor beta and down-regulated by hypoxia. Catenin (cadherin-associated protein), beta 1 (CTNNB1, 0.8-fold down-regulation; Figure 5D), protein encoded by this gene is part of a complex of proteins that constitute adherens junctions (AJs). AJs are necessary for the creation and maintenance of cell layers by regulating cell growth and adhesion between cells.

The A-kinase anchor protein 5 (AKAP5), a member of the AKAP family and also known as AKAP75 or AKAP79. AKAP5 is predominantly expressed in cerebral cortex and may anchor the PKA protein at postsynaptic densities (PSD) and be involved in the regulation of postsynaptic events. It can bind to the RII-beta regulatory subunit of cAMP-dependent protein kinase (PKA), and also to protein kinase C and the phosphatase calcineurin [32]. AKAP5/AKAP79 is present in the lipid raft of stimulated KG1 cells [33]. Down–regulation of AKAP5 in HBMVEC by TGRL lipolysis products suggests calcineurin-dependent NFAT signalling may involve. Retinoic acid receptor, beta (RARβ), a member of the thyroid-steroid hormone receptor superfamily of nuclear transcriptional regulators was increased 1.3-fold but was not significant. It binds retinoic acid, which mediates cellular signalling in embryonic morphogenesis, and cell growth and differentiation. This protein limits growth of many cell types by regulating gene expression. The mechanism associated with modulation of ERK 1/2 and JNK activation and depends on stimulation of RARβ [34].

**Figure 6:**
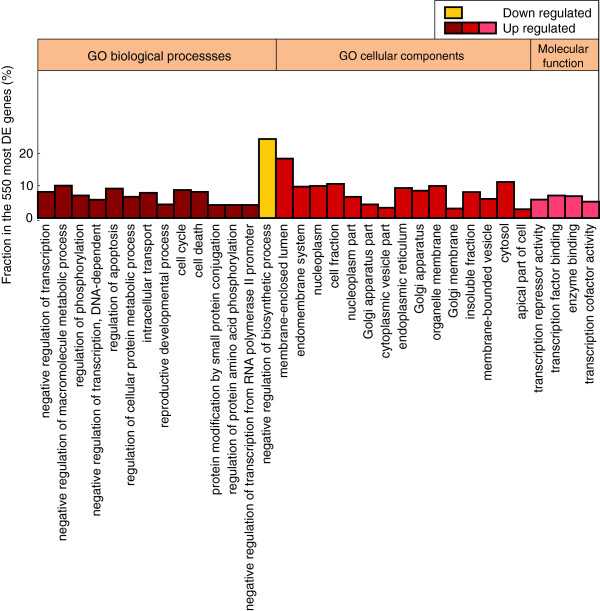
**Functional analysis of enriched differentially expressed clusters.** Percent of genes that belong in a significantly enriched functional annotation category, out of the 550 top DE genes. Cluster enrichment for GO biological processes, cellular components and molecular function is presented. Functional classes are indexed by increasing p-values: 8e-9 (negative regulation of transcription) to 3e-4 (negative regulation of biosynthetic process) for GO biological processes, 6e-8 (membrane-enclosed lumen) to 3e-3 (apical part of cell) for GO cellular components and 1e-6 (transcription repressor activity) to 3e-4 (transcription cofactor activity) for GO molecular function.

To gain a better insight on the biological processes, cellular components and molecular functions that are affected by the differentially expressed genes, we performed a functional analysis by using the DAVID toolbox (see Methods) [35,36]. As shown in Figure 6, negative regulation of transcription and biosynthetic processes were highly enriched, as well as the positive regulation of apoptosis and intracellular signalling. Not surprisingly, given the implicated metabolic compounds, membrane-related components and various organelles were the most differentially expressed genes were localized. In agreement with our analysis above, the functional enrichment analysis shows a clear differential regulation of transcriptional units, including transcription factor and cofactor binding, repressor activity and enzyme binding.

## Discussion

The most significant discovery of this study is the robust and rapid induction of genes encoding transcription factors, inflammation, and apoptosis. More specifically, HBMVEC treated with TGRL lipolysis products encode transcription factor ATF3-related genes in the MAP Kinase pathway associated with AP-1 signalling. This, then, stimulates an array of pro-inflammatory and pro-apoptotic genes that effect endothelial cell injury. ATF3 can be induced by stress and growth factors in mammalian cells, and is thought to play an important role in the cardiovascular and nervous system. However, little is currently known about how the induction of ATF3 is regulated, except that the JNK pathway is involved. Previous studies have shown up-regulation of ATF3 to act as either pro-inflammatory [37] or anti-inflammatory [38-40] and it was more recently linked to Alzheimer’s disease through a system-level approach [41]. ATF3 belongs to the ATF/cyclic AMP responsive element binding (CREB) family of transcription factors, characterized by a basic region leucine zipper (bZip) motif. The basic region is necessary for binding to the ATF/CRE promoter and the leucine zipper element for homo-and hetero-dimerization with other bZip proteins to differentially regulate transcription. The dimeric state of ATF3 gives us clues as to the ultimate effects of ATF3 on inflammation. As a homo-dimer, ATF3 acts as a transcriptional regulator inhibiting expression of pro-apoptotic molecules. Alternatively, ATF3 can form a heterodimer with activated c-Jun that enhances transcription of stress response genes [42]. The transcription of ATF3 as a key response gene after treatment for 3 hr with TGRL lipolysis products demonstrated that its induction was essential for the expression of a subset of pro-inflammatory responses.

TGRL lipolysis products-induced signalling pathways involve in mitogen-activated protein (MAP) Kinase, TGF-β and p53 signalling. The four major MAP Kinase pathways, ERK, JNK, P38, and BMK/ERK5, perform activating phosphorylations of nuclear transcription factors. Of these, ERK has been shown to inhibit ATF3 expression while JNK activates ATF3 through transcriptional regulation [43]. JNK activation also results in phosphorylation of c-Jun, a component with ATF3 of a complex that binds AP-1 responsive promoter regions. As would be expected, c-Jun/AP-1, ATF2, ATF4, ATF6, CREB, Myc, C/EBPB, Erg-1, and other transcription factors have been shown to induce ATF3 expression [44,45].

Our study has shown that increased expression of ATF3 occurs early in response to stress by a mechanism requiring the related bZIP transcriptional regulator ATF4. ATF3 contributes to induction of the apoptosis-inducing protein DDIT3 transcriptional factor in response to TGRL lipolysis products at 3 hr. DDIT3, CHOP, or GADD153 protein functions as a dominant-negative inhibitor by forming heterodimers with other C/EBP members, such as C/EBP and is linked to diabetes [13,46,47]. Expression of transcriptional activators ATF4 and CHOP have been reported to be induced during ER stress [48]. Although ATF3, ATF4, and DDIT3 (CHOP) are co-ordinately expressed in response to ER stress, there are differences between these genes regarding the contribution of transcriptional control and the timing of induced expression.

Previous studies have shown up regulation of ATF3 to act as either pro-inflammatory [37] or anti-inflammatory [38-40]. The dimeric state of ATF3 gives us clues as to the ultimate effects of ATF3 on inflammation. As a homo-dimer, ATF3 acts as a transcriptional regulator inhibiting expression of pro-apoptotic molecules. Alternatively, ATF3 can form a heterodimer with activated c-Jun that enhances transcription of stress response genes [42]. A number of other biological molecules and pathways could contribute to the pro-inflammatory and pro-apoptotic effects of TGRL lipolysis products. In this study, mRNA levels of a number of inflammatory genes were markedly up-regulated in TGRL lipolysis products treated HBMVEC. These genes, including IL-8, CXCL-3, PRNP and NRIP1, are important cytokines, which participate in inflammatory cell mobilization and recruitment. Induction of plasma membrane PRNP suggested that PRNP interact with many other proteins and induced vascular inflammation. It was recently shown that unfolded protein response and ATF3 are associated with inflammation response related to atherosclerosis in endothelial cells [49,50]. Our previous studies have shown that TGRL lipolysis products activate stress response pathways that induce expression of multiple pro-inflammatory and pro-apoptotic genes leading to endothelial dysfunction [5,51].

TGRL lipolysis products-induced up regulation of HDAC9 suggested vascular inflammation in brain microvascular endothelial cells. Several studies elucidated that the effects of individual HDACs in vasculature. HDAC9 promotes angiogenesis and determines the angiogenic gene expression pattern of endothelial cells [52]. Although HDAC9 gene is significantly associated with large-vessel stroke risk in different population [53], it’s actions are not known in microvascular endothelial cells. Our study showed up-regulation of HDAC9, GADD45A and HMGB2 expression regulates cell cycle progression through p53 signalling by TGRL lipolysis products in HBMVEC. Additionally, up-regulation of RARβ and JNK, may play a role and further activate vascular inflammation and apoptosis in HBMVEC. Further studies are needed to validate the strength of our system biology analyses and to determine how TGRL lipolysis products regulate physiologically important cell signalling pathways. The marked increased expression of ATF3 suggested that upstream molecules, such as TGFβ suggest that the mitogen-activated protein kinase pathway activation in HBMVEC by TGRL lipolysis products is mediated through RARβ.

Lipolysis of TGRL produces an array of remnant lipoprotein particles, fatty acids, phospholipids, diglycerides, and monoglycerides [4]. Any and all of these lipids and lipoproteins could have either pro- or anti-inflammatory effects on the endothelial cells although our previous work suggest that it is the fatty acids that are the primary mediators of endothelial cell injury at high physiological or pathophysiological concentrations [54-56]. However, the broad activation of transcription factors and pro-inflammatory genes suggests that other components of TGRL lipolysis products also could be playing a part in addition to fatty acids.

The peak elevation in blood TGRL is about 3–4 hours after a meal and blood triglycerides can rise 0.3-fold to many-fold higher during the postprandial period. Lipolysis of TGRL by endothelial cell-associated lipoprotein lipase likewise peaks in the postprandial period thus producing more endothelial cell injury or lipotoxicity as a result of the release of TGRL lipolysis products directly on the endothelial cells. Rather than low-grade, chronic injury, endothelial cells are injured repetitively over the course of a day, which could be a more pathological form of injury.

The precise pathogenetic mechanisms associated with lipotoxic neurovasclular inflammation remain to be elucidated. These experiments confirm the results of our previous work showing that high physiological to pathophysiological concentrations of TGRL lipolysis products induce brain microvascular lipotoxicity resulting in endothelial cell injury and apoptosis. These effects could alter blood–brain barrier integrity allowing not only potentially injurious lipids access to the brain, but also enabling thrombosis to occur on the injured brain endothelium causing brain microinfarctions. Thus, neurovascular lipotoxicity could induce brain injury and resulting decline in cognitive function.

Our microarray data and system biology analysis give us possible clues involving cell signalling pathways in HBMVEC treated with TGRL lipolysis products. The systems biology approach that was taken in this study allows to identify statistically significant associations by using a consensus analysis of 4 different methods, as well as looking simultaneously at the transcriptional, protein-protein interaction and gene ontology networks. Using a supervised interaction inference methodology that exploits the experimentally identified interactions that are known so far, is likely to increase the true positive rate of our analysis when compared to unsupervised methods. An extension to this work is looking at how information propagates through the combined gene regulatory and signal transduction networks by applied information-based methodologies [57-60]. Our methodology will benefit substantially from increasing the number of biological replicates and the use of next-generation sequencing technologies (RNA-Seq) with appropriate coverage and depth, as in this study, mRNA of the control and treated groups were pooled (n = 3), while the confirmation by RT-PCR was done on individual samples (n = 3) in each of the groups. Despite this limitation, we were able to confirm approximately 50% of the genes that were predicted by our computational analyses to be implicated in this phenomenon.

## Conclusions

Our systems biology approach demonstrated a robust and rapid induction of genes encoding transcription factors, inflammation, and apoptosis. Specifically, HBMVEC treated with TGRL lipolysis products encode transcription factor ATF3-related genes in the MAP kinase pathway associated with AP-1 signalling, which stimulates an array of pro-inflammatory and pro-apoptotic genes that effect endothelial cell injury. These experiments confirm the results of our previous work and demonstrate the complex, parallel, and multifaceted aspects of cell signalling that high physiological to pathophysiological concentrations of TGRL lipolysis products induce in brain microvascular lipotoxicity resulting in endothelial cell injury and apoptosis. Future studies are needed to show if these neurovascular pathophysiological processes are associated with the development of cognitive impairment.

## Methods

Human TGRL isolation: Postprandial blood samples were obtained 3.5 h after consumption of a moderately high-fat meal, which generally corresponds to the peak elevation in plasma triglyceride concentrations. Triglyceride-rich lipoproteins (TGRL) were isolated from human plasma at a density of less than 1.0063 g/mL following an 18 h centrifugation at 40,000 rpm in a SW41 Ti swinging bucket rotor (Beckman Coulter, Sunnyvale, CA) held at 14°C within a Beckman L8-70 M (Beckman) ultracentrifuge. The top fraction (TGRL) was collected and dialyzed in Spectrapor membrane tubing (mol wt cut off 3,500; Spectrum Medical Industries, Los Angeles, CA) at 4°C overnight against a saline solution containing 0.01% EDTA. This protocol was approved by the Human Subjects Review Committee at the University of California Davis.

### Cell culture and lipid treatments

Human brain microvascular endothelial cells (HBMVEC) (passage 6, Cell Systems, Kirkland, WA) were cultured in CSC Complete Medium which includes 10% serum (4Z0-500) (Cell Systems, Kirkland, WA) under an atmosphere of 5% CO2: 95% air at 37°C. Lipoprotein lipase (LpL) (L2254) was purchased from Sigma, St. Louis, MO. 3´ IVT Express Kit was purchased from Affymetrix, Santa Clara, CA. Cells were exposed for 3 hr to the following conditions, media, TGRL (150 mg/dL =1.5 mg/mL), lipoprotein lipase (LpL) (2 U/mL), and TGRL lipolysis product (TGRL (150 mg/dL) + LpL (2 U/mL)). The final concentration of TGRL, LpL and TGRL lipolysis products were diluted in media and pre-incubated for 30 minute at 37°C prior to application. After incubation, cells were washed with cold PBS and further process for RNA extraction.

### RNA extraction and synthesis of biotin-labeled RNA

Total RNA was extracted from Media, LpL, TGRL, and TGRL lipolysis products in HBMVEC in 6-well plate using RNeasy Mini Kit (Qiagen) including the DNA digestion step as described by the manufacturer. Microarray experiments were performed with pooled RNA isolates from each treatment group.

### GeneChip analyses

GeneChip analyses of the pooled total RNA samples (n = 3 per group for Media, LpL, TGRL, and TGRL lipolysis products treatments) were performed as previously described [5,61]. A 200 ng aliquot of total RNA from each pooled sample was reverse-transcribed, followed by aRNA Amplification, reverse transcription to synthesize first-strand cDNA, second-strand cDNA Synthesis, in vitro transcription to synthesize labeled aRNA, purification and fragmentation of aRNA as described in the Affymetrix 3´ IVT Express Kit protocol (Affymetrix, Santa Clara, CA). The fragmentation of labeled aRNA samples were hybridized to Human Genome U133A 2.0 Array high-density oligonucleotide arrays with ~22,000 probe sets representing 14,500 well-characterized human genes (Affymetrix). The hybridization, washing, labeling, and scanning of the GeneChips were performed as described in the Affymetrix protocols by the Microarray Core Facility in the UC Davis Genome and Biomedical Sciences Facility. The microarray dataset was deposited to Gene Expression Omnibus (GEO) with accession ID GSE57526.

### Validation of changes in mRNA expression by quantitative RT-PCR (qRT-PCR)

Since GeneChip analysis was done on pooled samples from each experimental group, confirming qRT-PCR analyses were performed on individual aliquots of total RNA samples from each treatment replicate. The purpose of these analyses was to evaluate the reliability of GeneChip data and develop statistical data to validate the changes suggested by the GeneChip assay of pooled RNA samples as our previous study as two assays may not match quantitatively but they always match qualitatively. Two analytical procedures may contribute to these discrepancies. 1) Total RNA samples used for the PCR assay are different from those used for the microarray assays. 2) The method of hybridization and detection for the two assays are different; the microarray assay utilizes hybridization of fragmented cRNA generated by Affymetrix protocols, whereas the RT-PCR utilizes amplification of DNA fragments using specific primer sets designed by a different method.

An aliquot equivalent to 5 μg of total RNA extracted from each sample was reverse-transcribed to obtain cDNA in a final volume of 20 μl solution consisting of buffer, oligo-dT primer, DTT, dNTPs, and Superscript-III reverse transcriptase (Invitrogen). RT-PCR with SYBR as fluorescent reporter was used to quantify the expression of selected genes identified by GeneChip analysis. Specific primers (Additional file 1: Table S1) were designed with Primer Express 1.0 software (Applied Biosystems) using the gene sequences obtained from Affymetrix Probeset IDs. Reactions were carried out in 384-well optical plates containing 25 ng RNA in each well. The quantity of applied RNA was normalized by simultaneously amplifying cDNA samples with glyceraldehyde-3-phosphate dehydrogenase (GAPDH)-specific primers. The transcript levels were measured by real-time RT-PCR using the ABI Vii7 Sequence detection system (PE Applied Biosystems, Foster City, CA). The PCR amplification parameters were: initial denaturation step at 95°C for 10 min followed by 40 cycles, each at 95°C for 15 s (melting) and 60°C for 1 min (annealing and extension). A comparative threshold cycle (Ct) method [62] was used to calculate relative changes in gene expression determined from real-time quantitative PCR experiments [Applied Biosystems user bulletin no. 2 (P/N4303859)]. The Ct, which correlates inversely with the target mRNA levels, was measured as the cycle number at which the SYBR Green emission increases above a preset threshold level. The specific mRNA transcripts were expressed as fold difference in the expression of the specific mRNAs in RNA samples from the TGRL lipolysis-treated cells compared with those from the control-treated cells.

### Analysis and differential expression

In conjunction with the AFFY Bioconductor package [63], we used several methods to extract the expression profiles and normalize the data. These include the BGX8 and mmgMOS [64] algorithms that take into account both perfect-match (PM) and mismatch (MM) probes to increase the statistical significance of the findings. To identify the differential expression (DE) profiles among the four conditions, we used the PUMA [65], PPLR [66] and iPPRL [67] packages. In addition we considered the popular MAS5 and RMA methods, although the later do not take into account the MM probes, which decrease the confidence level of the results.

### Functional and PPI networks

We used probabilistic Bayesian methods via an open-source package that uses Gene Ontology (GO) and KEGG annotations to create a functional gene network from microarray expression profiling data [68]. We integrated the expression-derived relationships with the known protein-protein interaction (PPI) network that was extracted with BIONET Bioconductor package [6] and by using an established base network (Interactome Library [7]). We then used the DAVID (The Database for Annotation, Visualization and Integrated Discovery) tool [35,36] to extract functional annotation terms enriched in the top 550 differentially expressed genes with respect to the control. We analyzed up- and down- regulated genes independently. Gene Ontology (GO) terms such as cellular components, molecular functions and biological processes were considered. We select the number of significant functional annotation terms produced from DAVID for each gene set based on the following criteria: (a) we only consider terms which include at least 2.5% of the gene set and (b) we consider terms to be statistically significant if they have a Benjamini-adjusted p-value less than 0.05. Highly related annotation terms (k > 0.9) are grouped by the term with the most genes and by their parent terms. Only terms in the GO FAT category are selected in order to filter out the very broad ones (Figure 6).

### Gene regulatory network inference

After literature curation, we created a dataset of all known transcription factor (TF)-gene interactions in human cells, with more than 4,000 experimentally validated interactions. Microvascular endothelial cell specific experiments [69-75] account for less than 5% of the connections we currently use in the training phase and are not adequate for training, as this would radically limit the scope of the method in finding new connections than the already established ones. Thus, the whole set of interactions was used as the training set for supervised learning through SIRENE [8], which uses support vector machines (SVM) [76] to classify genes to specific transcription factor bins, based on how related their expression pattern is to other genes that are known to be regulated by a specific TF. The resulting condition-specific Gene Regulatory Network (GRN) associated with TGRL lipolysis treatment was further enhanced by the regulatory predictions derived (repression/activation) by using Pearson correlation coefficient (PCC) on the expression profiles of the participants in each of the inferred connections regulator-target pair.

### Ethics

No human subjects were used for this study.

## Competing interests

The authors declare that they have no competing interests.

## Authors’ contributions

JCR and HHA initiated the study. HHA prepared the samples for analysis of microarray and the quantitative RT-PCR. AT performed the bioinformatics analysis. IT supervised all aspects of the computational analysis. HHA, NT, JCR and IT wrote the manuscript. All authors read and approved the final manuscript.

## Supplementary Material

Additional file 1 **Supporting material. Table S1.** Primer Sequences. Click here for file
