# Supplementary material for: A systems biology analysis of brain microvascular endothelial cell lipotoxicity
Source: BMC Syst Biol. 2014 Jul 4;8:80. doi: 10.1186/1752-0509-8-80 (PMC4112729; doi:10.1186/1752-0509-8-80)
Supplement: Additional file 1 — Supporting material. Table S1. Primer Sequences. [file 1752-0509-8-80-S1.docx]

# A Systems Biology Analysis of Brain Microvascular Endothelial Cell Lipotoxicity

Hnin H. Aung ^1*^, Athanasios Tsoukalas ^2,3*^, John C. Rutledge ^1§^, Ilias Tagkopoulos ^2,3§^

^1^ Division of Cardiovascular Medicine, Department of Internal Medicine, University of California, Davis, CA, 95616, USA

^2^ UC Davis Genome Center, University of California, Davis, CA, 95616, USA

^3^ Department of Computer Science, University of California, Davis, CA, 95616, USA

* These authors contributed equally in this work

**SUPPORTING MATERIAL**

We provide the primers that were used for the qRT-PCR validation analysis. The oligonucleotide sequences for each primer sequence were obtained from Affymetrix data base using the probe set IDs. The primers were custom prepared and used as described in the Methods section of the main manuscript.

**Supplementary Table 1. Primer Sequences**

| Gene | Primer sequence (5' - 3') |
| --- | --- |
| GAPDH | Sense-CACCAACTGCTTAG |
|  | Antisense-TGGTCATGAGTCCT |
| ATF3 | Sense-TTCTCCCAGCGTTAACACAAAA |
|  | Antisense-AGAGGACCTGCCATCATGCT |
| ATF4 | Sense-GTGGCATCTGTATGAGCCCA |
|  | Antisense-GGCTGTGCTGAGGAGACCC |
| DDIT3 | Sense-AGAGTGGTCATTCCCCAGCC |
|  | Antisense-CTTTCTCCTTCATGCGCTGC |
| KLF4 | Sense-ACTGGAAGTTGTGGATATCAGGG |
|  | Antisense-CTCCCCCAACTCACGGATATAA |
| PPARD | Sense-GCCAGGAGAATCGCTTGAAC |
|  | Antisense-TGCAGTGGTGGGATTTTGG |
| SP3 | Sense-TTCCAAAAACCATGTCTCCCA |
|  | Antisense-TGGTAATTTACATGATGGCTTTTAAGG |
| IL8 | Sense-CCTTTCCACCCCAAATTTATCA |
|  | Antisense-TGGTCCACTCTCAATCACTCTCAG |
| CXCL3 | Sense-TAGGGACAGCTGGAAAGGGA |
|  | Antisense-ACCCTCGTAAGAAATAGTCAAACACAT |
| PRNP | Sense-GGAAACCCTTTTGCGTGGT |
|  | Antisense-GAAACGATTCAGTGCACATTGTAAG |
| NRIP1 | Sense-TGTGATTACCTGCTGCATGAAAA |
|  | Antisense-CACAGATGCACAGGGTCCC |
| HDAC9 | Sense-TTTAATCAAGAAACTACCTGGAACCA |
|  | Antisense-ATTTAAGTCCAGCTTTCCTTTTCACT |
| GADD45A | Sense-GGCCCGGAGATAGATGACTTT |
|  | Antisense-CCTTCTTCATTTTCACCTCTTTCC |
| HMGB2 | Sense-GGCAATTATTTTGCTAAGAATGTGAAT |
|  | Antisense-AAGCTAGTATTGAGCTGCACTTGAAT |
| MDM2 | Sense-TGTTACCCAGGCTGGAGTGC |
|  | Antisense-CAGAGCTTGCAGTGAGCCAA |
| UGDH | Sense-TTGATACTTGTGCTCTGCTGAGAAT |
|  | Antisense-CCTGGGATGTTTAATGCAAACTG |
| CTNNB1 | Sense-TGTTATTTGGAACCTTGTTTTGGA |
|  | Antisense-TTGGGATAAAAGGCAACTGGTAA |
| AKAP5 | Sense-GAAACAGACCATCAGCAGCCA |
|  | Antisense-TGCAGGTAAAGGAGGAACATCAG |
| RARβ | Sense-ATGCTGGATTTGGTCCTCTGA |
|  | Antisense-GCTGGTTGGCAAAGGTGAAC |
